# The Deliverances of the Retina

**Published:** 1882-07

**Authors:** Geo. E. Blackham

**Affiliations:** Dunkirk, N. Y.


### BUFFALO

### Medical and Surgical Journal.

Vol. XXI. JULY, 1882. No. 12.

### Original Communications.

### The Deliverances of the Retina.

By Geo. E. Blackham, M. D., F. R. M. S., Dunkirk, N. Y.

In the April number of this Journal is published the very interesting address, with the same title which I have adopted, delivered by Dr. F. W. Abbott before the Alumni Association of the Medical Department of the University of Buffalo, at its last annual meeting.

In this paper, if I have read it aright, Dr. Abbott lays down as facts established by the latest researches of foreign specialists and admitting of no discussion* the following:

1st. That the human retina is composed of ten layers.

2d. That the ninth layer is composed of rods and cones, which, though differing in shape, seem to be identical in structure, each being composed of an inner body striated, and an outer body which is perfectly transparent.

3d. That the tenth layer is the pigment layer which until recently has been considered a layer of the choroid, but is now considered by the latest authorities to be a true retinal layer and a very important one.

*Buffalo Med. and Surg. Journal, April, 1882, page 391.

4th. That the pigment layer sends processes down between the cones and rods filling the interspaces between them so that at the fovea each outer body of a cone is embedded in a mass of pigment which fits it as a glove fits a finger, and separates it from its neighbors on all sides by a dark opaque barrier.

5th. That the layer of rods and cones is the percipient layer of the retina.

6th. That the study of the physiology of the retina has to be entirely subjective. Microscope and test tube can not tell us what it does.

7th. That, if we compare the distance which separates the retinal pictures of two objects which can barely be distinguished with the thickness of the cones at the fovea, we shall find that they must be far enough apart to fall on two separate cones; and also that if two retinal pictures are so close together that they fall on one cone, we no longer get two sensations, we are no longer conscious of seeing two objects.

With these facts as postulates Dr. Abbott proceeds to the theoretical part of his address which he very frankly says may admit of considerable discussion.

He rejects as erroneous the theory that the rods and cones are directly sensitive to light, and the light waves in passing through the cones directly irritate them, and offers in place of it another theory which seems to him to explain the facts much more satisfactorily, which he does not claim as original with himself, but for which he gives credit to Prof. Fick's article on the eye in the *Handbuch der Physiologie*, edited by Prof. Hermann, of Zurich:

"This theory is that the rays of light do not irritate the rods and cones directly, as they pass through them, but that these light rays, after passing through the transparent layers of the retina, without doing any work, set up a molecular motion in the pigment layer, and this molecular motion of the pigment layer beating against the cones and rods irritates them."

This theory he supports by the following arguments.

1st. That the cones which are said to have been irritated by light rays are perfectly transparent, *i. e.*, they allow ninety-nine per cent. of the light to pass through them unchanged.

2d. That while it is impossible to deny with mathematical certainty that one per cent. of the light is sufficient to irritate the cone and give sensation, yet, since faint starlight suffices to excite the retina, while sunlight or the dazzling glare of electricity is necessary to print a picture on the photographer's plate, analogy and reason teach us that it is impossible that ninety-nine per cent. of the faint starbeams have gone to death or been uselessly absorbed in the pigment, and that one per cent. only has been utilized in doing the work for which the retina was intended.

3d. That if this theory is true, we are not obliged to attribute special properties to the optic nerve fibres and rods and cones, which isolate them from the rest of the nervous system.

When a theory is offered as new by an authority of sufficient standing to make it worthy of investigation, two questions naturally arise.

1st. Is it new?

2d. Is it true?

The second, of course, being the much more important one.

That this theory is not new will, I think, be evident to any one who will read through the chapter on vision in Draper's Human Physiology, published by Prof. John W. Draper, M. D., LL. D., of New York—7th Edition, 1865—eighteen years ago. Dr. Draper there shows, that assuming the retina to be perfectly or very nearly transparent, it is unfitted for the reception of images; that the light rays pressing through the retina are absorbed by the pigment layer, and thereby raise its temperature in varying degrees, that is to say, set up a molecular motion, which is recognized by the rods and cones (or as he calls them the club-shaped particles of Jacob's membrane). Prof. Draper by no means ties himself to the calorific expression of his hypothesis, but says distinctly:

"It remains now to add that this is only one manner of looking at the thing. According as our hypothesis of the nature of light, of its relations to heat, and of its manner of establishing chemical changes may be, the special explanation we give of the functions of the eye will differ; yet there is such a relationship among these hypotheses that we can, without any difficulty, convert an explanation derived from one into an explanation derived from another. It really comes to little more than a translation of phraseology. I have found the calorific hypothesis convenient. * * * Yet, with almost equal convenience, the function might have been treated otherwise, viewing light as arising from ethereal undulations, the additional advantage then being obtained of establishing a parallel between the action of the organ of sight and that of hearing. Or, in like manner, the case might have been viewed in its purely chemical aspect, photographically, as it might be said. * * * But this, again, amounts only to a different mode of stating the same effect, since, as I have shown, all chemical changes accomplished in material substances are occasioned by the establishment of vibratory motions therein, and Ampere has already demonstrated that all the phenomena of heat may be explained upon the doctrine of the vibrations of the constituent molecules of bodies."*

It is evident then that Dr. Draper announced at least eighteen years ago this theory, that the rays of light passed through the layer of rods and cones were absorbed by the pigment layer—spent their force in setting up molecular motions in that layer—which motions were perceived by the layer of rods and cones which he describes as tactile organs, and further that the molecular motions set up in the pigment layer might be described in terms of heat, light or chemical activity as might be most convenient. Thus much as to the newness of this theory and its transatlantic origin. Next as to its truth.

Two methods, at least, are open to those who wish to disprove a theory.

---

*Draper's Physiology—7th Ed.—Harper Bros., N. Y., page 399.

1st. To show that it is inconsistent with the postulates on which it claims to be founded, and

2d. To show that the postulates are not in themselves correct.

1st. Then, this theory is inconsistent with the postulates laid down by Dr. Abbott, in that, in order to distinguish two objects or points as separate, their retinal images must fall on two separate cones, while if so close as to fall upon one cone only we get only one image. This shows that it is upon the cones themselves and not in the intermediate pigment that the images must be formed. Further, if the images were formed and the molecular disturbance which originates vision set up in the masses of pigment which separate the cones, more than one cone would necessarily be irritated by each mass of pigment thus thrown into molecular motion.

If we stand up a number of sticks in water and drop a stone among them so as not to touch any of them the impulses thus conveyed to the particles of water will spread in every direction, as shown by the circular wave on the surface, and beat upon at least four of the sticks. Thus a molecular motion set up in the pigment between the cones would spread so as to affect, at least, four cones, and the result, according to Dr. Abbott's postulates would be four sensations or images instead of one.

It is thus shown that this theory is not consistent with the postulates upon which it claims to be based.

Now as to the correctness of the postulates themselves:

1st. As to the question whether the retina is composed of nine or ten layers, and whether the tenth, the pigment layer, is properly assignable to the retina or choroid. It is to be remembered that this is largely a matter of nomenclature, and that these lines of demarcation are often artificial and imaginary, and while serving a purpose for description have no more real existence in nature than the equator or the tropics have on the material globe.

For reasons which I will give further on it seems better to me to consider the pigment layer as a part of the choroid, but it is no great matter after all.

The anatomical relations of the rods and cones to the pigment layer, as described by Dr. Abbott, may be accepted, but transparency of the rods and cones must be qualified. That they are practically transparent and colorless when viewed by ordinary light is easily demonstrated; that they (the rods at least) are not so at all times and under all circumstances, in fact, that such is not their normal condition, is also, though not so easily, demonstrable.

Herr Fr. Boll, in a communication to the Berlin academy (in 1876), announced the beautiful and, beyond doubt, important discovery, that the bacillary layer of the retina of all animals is in the living condition not colorless, as has been hitherto supposed, but of a purple red color. During life, says Boll, the proper color of the retina is continually being destroyed by the light which falls into the eye; it is restored in the dark, and after death remains only a few moments. Animals which have been kept in the light are therefore very unsuitable for demonstrating the living color of the retina, and the eyes of animals which have been exposed to the sun for a long time before death remain absolutely colorless. These facts illustrate the relation of the retinal color to light on the one hand, and to vital conditions on the other.*

Kuehne goes on to show that the process of bleaching and removal is continually going on in the normal eye during life. It is thus shown that the light does not normally pass through the retina without doing any work but that it finds employment in the bleaching process, and hence that the portion of it so consumed never reaches the pigment layer, and hence we are not confined to the two alternatives of belief that either the light is wasted or is expended in setting up molecular motions in the pigment layer, since it has enough to do in causing chemical

*Dr. W. Kuehne on "The Ph to Chemistry of the Retina" and on "Visual Purple"—English translated—Edited by Prof. Michael Foster, M. D., &c., London, 1878.

changes in the bacillary* layer which Dr. Abbott states "is the percipient layer of the retina."

This seems to answer fully the first and second arguments adduced by Dr. Abbott in support of the theory under discussion. As to the amount of light necessary to excite the retina and the photographer's plate respectively, it is only necessary here to note that there are different degrees of sensitiveness in eyes and in plates, and that some recent dry plates so far from requiring sunlight or the dazzling glare of electricity to print a picture on them, are perfectly responsive to the light of a common kerosene lamp, as a photo-micrograph now lying before me, the negative of which was taken by a few seconds' exposure to lamplight, amply testifies.

That images are really formed in the bacillary layer of the retina has been shown by Kuehne, who has succeeded in fixing them sufficiently to be exhibited after the eye has been removed from the body.

"Indeed, by confining rabbits in darkness for a length of time and then exposing them to a bright window crossed by bars, decapitating them in a room lighted only by a sodium flame, and treating the retina by a solution of alum, * * * a picture or optogram can be developed and fixed upon the retina and preserved for future study. Such a picture is given in the diagram (fig. 4) copied from the New York Medical Journal, March, 1881, and taken by Dr. Ayers who worked with Prof Kuehne in his laboratory."*

If the percentage of the light rays which do not pass through the retina without doing any work are sufficient to produce these photographic effects it may well be believed that they are sufficient to originate the sensation of vision.

As to the final argument that this theory, if true, relieves us from the necessity of attributing special properties to the optic fibres and the rods and cones, I can not see that this is any

*Noyes, on "Diseases of the Eye." Wm. Wood & Co., New York, December, 1881, pp. 5 & 6. See also Foster's Physiology, 3d Ed. MacMillan & Co., New York, 1880, pp. 533 to 538. Also Kuehne, l. c.

advantage. I was under the impression that this very specialization of function was one of the distinguishing marks of a high degree of development or exalted position in the scale of animate existence. Not only is this true, but, according to Kuehne, the possession of these special properties brings the terminal fibres of the optic nerve into harmony with other nerves of special sense which are peculiarly sensitive to chemical action viz.: the nerves of taste and smell. It seems then neither the postulates upon which this theory is founded nor the arguments by which it is supported will bear critical examination in the light of late researches.

We may conclude then that the human retina is composed of layers; the ninth, being the bacillary or rod and cone layer, is continually being stained purple by the pigment layer* and bleached by the action of light; that is necessary for the images to be found in this bacillary layer, that visual impressions may be received, therefore it follows that these images must not be formed in the pigment layer. The pigment layer being the source of the sensitizing material or materials (visual purple, etc.,) may be compared to the photographer's sensitizing bath (in the wet process), and the bacillary layer to the plate from which the image is continually being effaced by the resensitizing process. That ninety-nine per cent. of the light does not pass through the retina without doing any work to be absorbed by the pigment, but enough is used up in the retina to produce images by bleaching, and hence enough to imitate visual sensations. Therefore while Dr. Draper, eighteen years ago, may have been justified (before the discovery of the color of the living retina) in adopting the theory that it is in the pigment layer, and not the bacillary layer, that images are formed and visual sensations initiated, we are not at present, and in the light of recent investigations, justified in accepting that theory.

*The pigment layer is thus seen to be a depot or magazine, while the visual purple, derived from the abundant blood supply of choroid, is stored up till wanted to resensitize the bleached portions of the retina; it therefore seems better to consider it as a layer of choroid which is essentially a vascular and pigmentary structure than of the retina which is essentially a nervous and sensitive structure, but as I have said this is largely a matter of convenience of nomenclature.

On the other hand we are not justified in rushing to the conclusion that vision depends *solely* upon the bleaching of visual purple which has been absorbed by the bacillary layer of the retina, for Kuehne and others have shown that vision is possible without visual purple. There may be, and probably are, other substances concerned in the photo-chemistry of the retina. What has been shown is that the changes, whatever they are, take place chiefly in layer of rods and cones and not in the pigment layer.

Happily, though we are not at present prepared to formulate a complete theory of vision, we are constantly accumulating information on which to found one. The microscope has revealed to us the structure of the rods and cones, and the fact that light does produce an effect on this structure (in addition to the bleaching). The test tube has helped us to a knowledge of the chemical constitution of visual purple, and its reactions under the influence of various kinds of light and with various reagents. The study of the physiology of the retina has ceased to be "entirely subjective," and has thus been removed from the nebulous domain of metaphysics to the firmer if more prosaic region of physical science.

